# Frontal variant of Alzheimer's disease with asymmetric presentation mimicking frontotemporal dementia: Case report and literature review

**DOI:** 10.1002/brb3.1548

**Published:** 2020-01-27

**Authors:** Cheng‐Hsuan Li, Sung‐Pin Fan, Ta‐Fu Chen, Ming‐Jang Chiu, Ruoh‐Fang Yen, Chin‐Hsien Lin

**Affiliations:** ^1^ Department of Neurology National Taiwan University Hospital Taipei Taiwan; ^2^ Department of Neurology National Taiwan University Hospital Hsinchu Taiwan; ^3^ Graduate Institute of Brain and Mind Sciences College of Medicine National Taiwan University Taipei Taiwan; ^4^ Graduate Institute of Biomedical Engineering and Bioinformatics National Taiwan University Taipei Taiwan; ^5^ Graduate institute of Psychology National Taiwan University Taipei Taiwan; ^6^ Department of Nuclear Medicine National Taiwan University Hospital Taipei Taiwan

**Keywords:** behavior variant of frontotemporal dementia, beta‐amyloid, biomarkers, frontal variant of Alzheimer's disease, positron emission tomography, tau

## Abstract

**Introduction:**

Frontal variant of Alzheimer's disease (fvAD) is a rare nonamnestic syndrome of Alzheimer's disease (AD). Differentiating it from behavior variant of frontotemporal dementia (bvFTD), which has implications for treatment responses and prognosis, remains a clinical challenge.

**Methods:**

Molecular neuroimaging and biofluid markers were performed for the index patient for accurate premortem diagnosis of fvAD. The clinical, neuroimaging, and biofluid characteristics of the patient were compared to those reported in previous studies of fvAD from 1999 to 2019.

**Results:**

A 66‐year‐old man presented with progressive executive dysfunction, personality and behavioral changes, and memory decline since age 59. He had no family history of neurodegenerative disorders. A stimulus‐sensitive myoclonus was noted over his left upper extremity. Neuropsychological assessment revealed moderate dementia with a Mini‐Mental State Exam score of 10/30 and compromised executive and memory performance. Brain imaging showed asymmetrical atrophy and hypometabolism over the right frontal and temporal areas, mimicking bvFTD. However, we observed increased tau depositions based on ^18^F‐labeled T807 Tau PET in these areas and diffusely increased amyloid deposition based on ^11^C‐labeled Pittsburgh compound B positron emission tomography (PET). Plasma biomarker measures indicated an AD profile with increased Aβ1‐42 (18.66 pg/ml; cutoff: 16.42 pg/ml), Aβ1‐42/Aβ1‐40 ratio (0.45; cutoff: 0.30), total tau (29.78 pg/ml; cutoff: 23.89 pg/ml), and phosphorylated tau (4.11 pg/ml; cutoff: 3.08 pg/ml). These results supported a diagnosis of fvAD.

**Conclusions:**

Our results with asymmetrical presentations extend current knowledge about this rare AD variant. Application of biofluid and molecular imaging markers could assist in early, accurate diagnosis.

## Graphical abstract

A 66‐year‐old man presented with progressive executive dysfunction, personality and behavioral changes, memory decline, and asymmetric motor symptoms. Brain molecular imaging revealed amyloid and tau retention, which suggest the diagnosis of frontal variant of Alzheimer's disease.

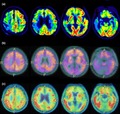

## 1 INTRODUCTION

Alzheimer's disease (AD) is the most prevalent neurodegenerative disease worldwide, typically characterized by progressive episodic memory loss at onset, followed by impairment in other cognitive domains (Dubois et al., 2014). Its distinct pathology consists of a dual proteinopathy involving extracellular neuritic amyloid‐β (Aβ) plaques and intracellular aggregates of hyperphosphorylated tau (p‐tau) as neurofibrillary tangles (NFTs) (Dubois et al., 2014). However, in some atypical presentations of AD, nonamnestic symptoms predominate in the early disease course. As proposed by the International Working Group diagnostic criteria for AD (IWG‐2 criteria) (Dubois et al., 2014), specific phenotypes of atypical AD include posterior cortical atrophy, logopenic primary progressive aphasia, Down's syndrome variant, and frontal variant of AD (fvAD) (Alladi et al., 2007). Among these nonamnestic syndromes, fvAD is quite uncommon compared to other subtypes of AD (Alladi et al., 2007; Mendez, Lee, Joshi, & Shapira, 2012). Patients with fvAD often display prominent dysexecutive and behavioral problems at presentation, supposedly because of selective neurodegeneration in frontal control networks (Wolk, 2013). The clinical presentation may lead to diagnostic conflation with behavioral variant frontotemporal dementia (bvFTD) because of many common symptoms, including behavioral disinhibition, apathy, perseverating behaviors, and executive dysfunction on cognitive testing. In contrast to AD, the pathological condition of bvFTD derives mostly from intracellular aggregates of tau protein or TAR DNA‐binding protein 43 and less frequently from intracellular fused in sarcoma inclusions (Perry et al., 2017).

Although patients with bvFTD are more likely to manifest asymmetrical presentations and neuroimaging findings, differentiating the fvAD and bvFTD based on clinical presentations remains a challenge, especially early in the disease course. Structural brain magnetic resonance imaging (MRI), perfusion single‐photon emission computed tomography, or metabolic fluorodeoxyglucose (FDG) positron emission tomography (PET) may not well differentiate fvAD from bvFTD because both syndromes involve deficits in the frontal lobes. With the recent advent of amyloid and tau PET molecular imaging (Rabinovici et al., 2007, 2011), and biofluid biomarkers of Aβ1–42, Aβ1–40, total and phosphorylated tau (Chiu et al., 2012, 2013), an integrated approach combining neuropsychological tests, molecular neuroimaging, and fluid biomarkers may assist clinicians in making an appropriate differential diagnosis between fvAD and bvFTD. As the treatment responses to cholinesterase inhibitors and disease course are better in patients with AD than with FTD, the clinical distinction between fvAD and bvFTD has implications for prognosis, treatment choices, and disease progression for affected patients (Li, Hai, Zhou, & Dong, 2015; Peters et al., 2015). Here, we present a patient who exhibited prominent frontal symptoms followed by amnesia with positive molecular neuroimaging and biofluid biomarkers for AD, leading to a diagnosis of fvAD.

## 2 CASE REPORT

A 66‐year‐old, right‐handed, college‐educated man was brought to our clinic because of progressive executive dysfunction, behavioral symptoms, and memory decline for around 7 years. He had retired from his job as a stock manager at age 58. By age 59, he started to present with progressive executive dysfunction in daily activities and was unable to properly carry out some household chores or organizing. In addition, he had become increasingly indifferent, less interested in hobbies, easily provoked by normal conversations and losing his temper toward his family. His language function was initially relatively preserved. Later, by age 62, he had symptoms of forgetfulness, such as repetitive questioning, missing appointments, or losing things, which became more prominent over time. He also became confused about routes that should have been familiar to him and got lost for many times. His condition rapidly deteriorated, and at age 65, he displayed more aggressiveness, with poor personal hygiene, and was frequently disoriented about time, space, and even about people. He also had hot temper and even had violent behaviors to the caregiver. His appetite and body weight did not change significantly.

On evaluation, there was no identified systemic disease, previous medication exposure, or a family history of dementia. Neurological examination was unremarkable. His gait appeared normal, without shuffling or stooped posture, except for a mildly decreased arm swing on the left side. He also had some myoclonic involuntary movements in his left upper extremity despite intact muscle strength and primary sensation. The Mini‐Mental State Examination (MMSE) revealed a total score of 10/30, with prominent impairment in orientation, attention/calculation, free recall, and pentagon copying. The complete neuropsychological test revealed significantly decreased executive function and moderate amnesia (Table 1). He failed to complete the trail‐making test, both parts A and B. Lexical fluency was significantly reduced, with an ability to name only six four‐legged animals in one minute. He showed poor performance in an assessment of judgment and abstract thinking, with perseveration in Luria's three‐step motor tests in both hands, and disinhibition in the go/no‐go test. Pathological palmomental and suck reflexes were observed. His total Frontal Assessment Battery score was only 1/18, indicating prominent frontal executive dysfunction. The language domain was relatively preserved, with fluent speech and fair performance in repetition and confrontation naming, but generally diminished speech contents. He also exhibited impairment in other cognitive domains, such as ideomotor and constructional apraxia.

**Table 1:** The results of the complete neuropsychological test of the index patient

Neuropsychological test	Index patient (scores/normal value or percentile)
MMSE	10/30^a^
Executive function	
FAB	1/18^a^ (<1 percentile)
Trail‐making test A	Cannot complete
Trail‐making test B	Cannot complete
Stroop: word	53^a^ (<1 percentile)
Stroop: color	45^a^ (1 percentile)
Stroop: color world	18^a^ (<1 percentile)
Frontal Behavioral Inventory	
Negative Behavior Score	18/36^a^ (normal range: <8/36)
Disinhibition Score	28/36^a^ (normal range: <8/36)
Wisconsin Card Sorting global score	108.1^a^ (normal range: <91)
Special perception function	
Judgement of line orientation	22/30 (45 percentile)
3D‐block construction model score	28/29 (40 percentile)
Memory function (FCSRT)	
Encoding	7/16^a^
Total Free Recall	0/48^a^
Total (Free + Cued) Recall	12/48^a^
Praxis	
Right hand	11/12
Left hand	7/12^a^
Language	
Visual naming	56/60 (54 percentile)
Token test	44/40 (10 percentile)
Aural comprehension	18/18 (>90 percentile)

Abbreviations: MMSE: mini‐mental state exam; FAB: frontal assessment battery; FCSRT: free and cued selective reminding test

a Pathological score, adjusted for age and education.

**Figure 1:**
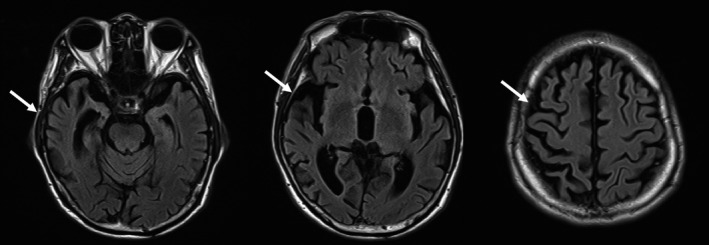
Brain MRI of the index patient. Brain MRI image, T2 FLAIR, showing atrophy in bilateral temporal, parietal, and frontal lobes, featuring asymmetric atrophy that is more severe in the right hemisphere (arrows). MRI, magnetic resonance imaging

**Figure 2:**
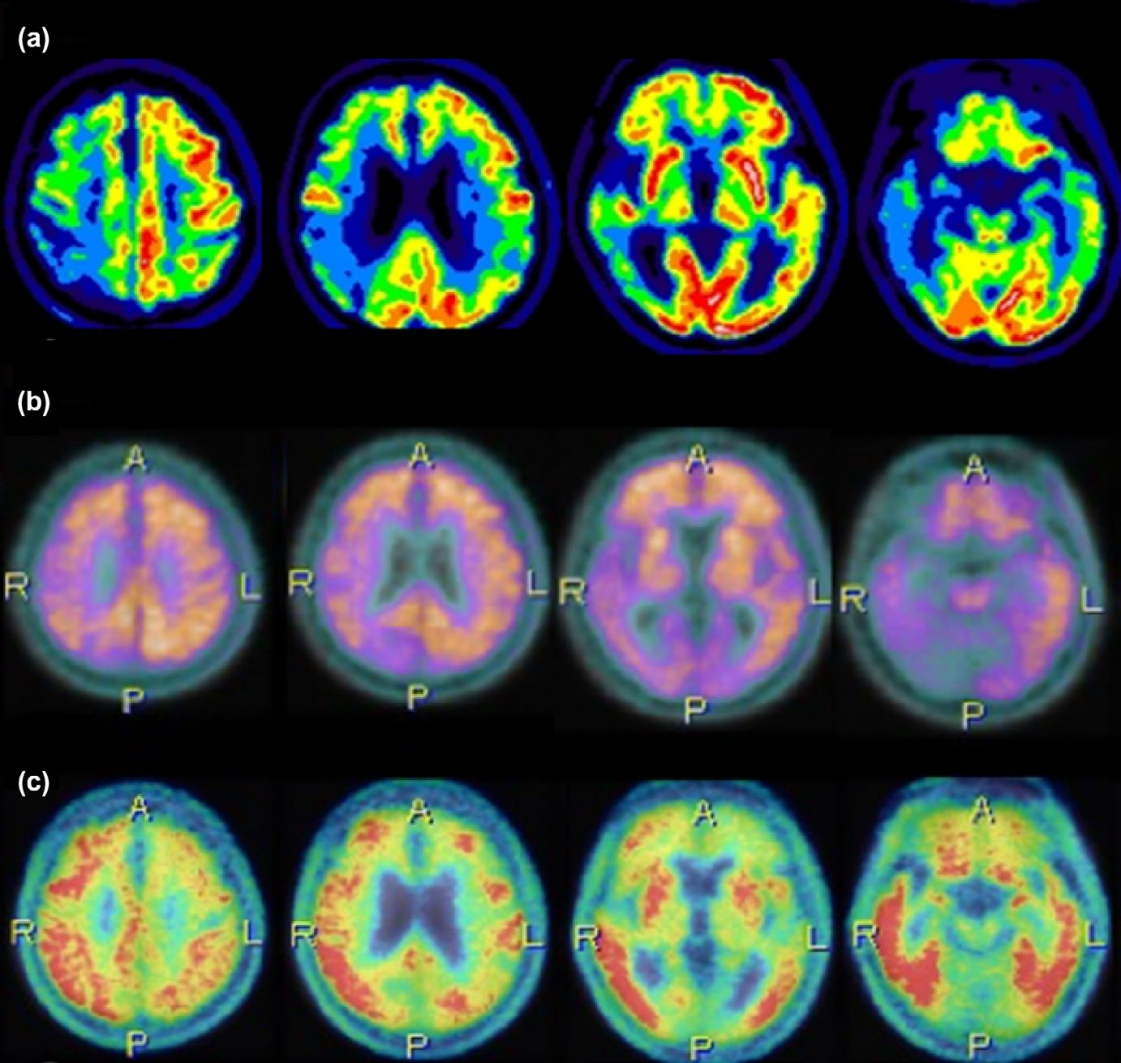
Brain FDG‐PET, ^11^C‐PiB amyloid PET, and ^18^F‐T807 tau PET image findings of the index patient. (a) FDG‐PET shows significant hypometabolism in the right frontal, parietal, occipital, and temporal regions and left lower parietal and superior temporal regions. (b) ^11^C‐PiB amyloid PET shows diffuse tracer retention in bilateral cerebral cortices, relatively lower in the right hemisphere and more obvious in frontal, parietal, precuneus, and anterior and posterior cingulate regions. (c) ^18^F‐T807 tau PET shows significant tracer retention in right frontal, parietal, and temporal regions and milder retention in the left parietal regions. The asymmetric topography was correlated with the hypometabolism pattern on FDG‐PET (a). FDG, metabolic fluorodeoxyglucose; PET, positron emission tomography; ^11^C‐PiB, ^11^C‐labeled Pittsburgh compound B

A comprehensive laboratory survey did not reveal any specific abnormality in serum glucose, lipid profile, liver and renal function, thyroid function, or vitamin B12 level. A CSF study showed no pleocytosis or elevated protein. Electroencephalography exhibited diffuse slow activities. The brain MRI revealed asymmetrical atrophy in the bilateral frontal, temporal, and parietal lobes that was more severe in the right hemisphere, without significant white matter change or vascular insult (Figure 1). The dopamine transporter imaging using Tc‐99m TRODAT‐1 perfusion single‐photon emission computed tomography displayed normal findings. However, ^18^F‐FDG PET revealed significant hypometabolism in the right hemisphere, especially in the right frontal, temporal, and parietal regions, along with relatively mild hypometabolism in the left lower parietal and superior temporal regions (Figure 2a). The ^11^C‐Pittsburgh compound B (^11^C‐PiB) amyloid PET imaging suggested diffuse amyloid retention in bilateral hemispheres (Figure 2b). The PiB‐PET imaging was processed and analyzed in PMOD software (version 3.7, PMOD Technologies Ltd., Zurich, Switzerland). The PET image was co‐registered to the T1‐weighted MR template and spatially normalized to the Montreal Neurological Institute (MNI) space. The automatic anatomic labeling atlas was applied for regions of interest on these spatially normalized images. The cerebellum was selected as the reference region to calculate the standard uptake value ratio (SUVR) of each region of interest (Table 2). We observed that the global cortical amyloid burden measured by the SUVR of a composite region (frontal, lateral temporal, lateral parietal, anterior and posterior cingulate, precuneus) was 1.72 (cerebellum as reference), higher than the cutoff value of 1.5 determined in a previous study (Villemagne et al., 2013). There was no significant difference of composite SUVR values between hemispheres (*p = *.125 by Wilcoxon signed‐rank test, Table 2). Regarding tau PET imaging using ^18^F‐T807 ligand (also called AV‐1451) (Chien et al., 2013; Shoup et al., 2013), tracer retention was diffusely elevated and markedly increased in the right frontal, right temporal, and bilateral parietal cortices (Figure 2c), which substantially correlated with the hypometabolism changes in the corresponding areas on FDG‐PET image. A composite SUVR using the same method as above yielded a value of 1.52 and showed asymmetrical increases in the right frontal region (Table 2).

**Table 2:** Regional tracer retention values in molecular imaging of ^11^C‐PiB amyloid PET and ^18^F‐T807 tau PET

Region	Right	Left	*p* value
SUVR of ^11^C‐PiB uptake reference to cerebellum			
Frontal	1.66	1.99	.125
Lateral temporal	1.34	1.77	
Parietal	1.54	2.01	
Lateral occipital	1.49	1.74	
Composite region	1.72		
SUVR of ^18^F‐T807 uptake reference to cerebellum			
Frontal	1.52	1.34	.250
Lateral temporal	1.77	1.62	
Parietal	1.43	1.57	
Lateral occipital	1.63	1.44	
Composite region	1.52		

Note

^11^C‐PiB: ^11^C‐labeled Pittsburgh compound B; PET: positron emission tomography; SUVR: standard uptake value ratio. The PiB uptake difference between hemispheres over these selected regions was not statistically significant under Wilcoxon signed‐rank test (*p* = .125). The T807 uptake difference between hemisphere was not statistically significant under Wilcoxon signed‐rank test (*p* = .250).

Considering his early‐onset cognitive decline, despite his lack of a family history of dementia, we applied a targeted next‐generation sequencing panel for detecting possible mutations in candidate genes related to AD and FTD, including *APP*, *PSEN1*, *PSEN2*, *MAPT*, *GRN*, *CHMP2B*, *TYROB*, *TREM2*, *PRNP*, *DCTN1*, and *NOTCH3*, all with negative results. The genetic analysis for G4C2 hexanucleotide repeats of *C9Orf72* was also negative. Plasma biomarker measures using an immunomagnetic reduction (IMR) assay (Chiu et al., 2012, 2013; Yang et al., 2018) indicated an AD profile with increased Aβ1‐42 (18.66 pg/ml; cutoff: 16.42 pg/ml), Aβ1‐42/Aβ1‐40 ratio (0.45; cutoff: 0.30), total tau (29.78 pg/ml; cutoff: 23.89 pg/ml), and phosphorylated tau (p‐T181 tau, 4.11 pg/ml; cutoff: 3.08 pg/ml) (Tables 3 and 4). Based on these findings, probable AD could be clinically diagnosed according to the 2011 National Institute on Aging and Alzheimer's Association criteria (McKhann et al., 2011), and fvAD could be diagnosed according to the IWG‐2 criteria (Dubois et al., 2014).

**Table 3:** Plasma biomarkers levels

Biomarker	Patient	Cutoff value
Aβ1‐42 (pg/ml)	18.66	<16.42
Aβ1‐40 (pg/ml)	41.20	<59.20
Aβ1‐42/Aβ1−40 ratio	0.45	<0.30
Total tau (pg/ml)	29.78	<23.89
p‐T181 tau (pg/ml)	4.11	<3.08
p‐T181 tau/total tau ratio	0.14	<0.14

Note

Plasma cutoff value was determined according to previous studies that differentiated patients with AD and normal controls (Chiu et al., 2012, 2013; Yang et al., 2018).

**Table 4:** Systematic literature review of demographic, clinical, neuroimaging, and biofluid marker data in patients with fvAD; data for age and disease duration were shown as mean ± *SD*

References	No. of cases	M/F	Age at examination, years	Disease duration, years	Education, years	MMSE	Compromised domains in NPT	Brain MRI	Molecular imaging	Biomarker assay	Pathology findings
Johnson et al. (1999)	3	2/1	71.7 ± 8.1	8.0 ± 2.0	13.3 ± 2.3	20.33	Executive function	N.A.	N.A.	N.A.	Amyloid (+), greater NFT (+) in frontal cortex compared to typical AD
Back‐Madruga et al. (2002)	10^a^	5/5	73.6 ± 9.6	2.2 ± 0.8	15.9 ± 2.5	22.2	Executive, memory, visuospatial function	N.A.	N.A.	N.A.	N.A.
Larner (2006)	2	2/0	54	N.A.	N.A.	19.5	Executive, memory, visuospatial function	Global atrophy	N.A.	N.A.	N.A.
Forman et al. (2006)	19^b^	10/9	60.3	8.9	15.8	20.1	Executive function, memory	N.A.	N.A.	N.A.	Widespread senile plaques (+) and tau (+)
Taylor, Probst, Miserez, Monsch, and Tolnay (2008)	1	1/0	66	3	N.A.	28	Attention	F‐T atrophy	N.A.	N.A.	Amyloid (+), greater NFT (+) in frontal cortex
Habek, Hajnsek, Zarkovic, Chudy, and Mubrin (2010)	1	1/0	56	4	N.A.	N.A.	N.A.	Global atrophy	N.A.	CSF Aβ1−42↓, total tau and p‐tau normal	Amyloid (+) and NFT (+) in frontal cortex by frontal lobe biopsy
Dickerson and Wolk, (2011)	27^a^	16/11	75.7 ± 8.8	N.A.	14.4 ± 3.8	23.7	Executive function > memory	F‐P atrophy	N.A.	N.A.	*N*.A.
Herrero‐San Martin et al. (2013)	2^b^	1/1	56	N.A.	N.A.	N.A.	Executive function	N.A.	N.A.	N.A.	AD pathology (+) affected the frontal lobes
de Souza et al., (2013)	8	7/1	63.5 ± 8.9	3.5 ± 2.4	10.4 ± 3.9	17.6	Executive function, memory	Global or F atrophy	N.A.	CSF Aβ1−42↓, total tau↑, p‐tau↑	N.A.
Mendez et al. (2013)	21^b^	18/3	69.3 ± 8.3	N.A.	16.3 ± 3.4	13.3	Executive function	N.A.	N.A.	N.A.	AD pathology (+)
Blennerhassett, Lillo, Halliday, Hodges, and Kril (2014)	6	4/2	68 ± 14	6.7 ± 3.2	N.A.	N.A.	Executive function > memory	N.A.	N.A.	N.A.	Amyloid (+), greater NFT (+) in frontal cortex compared to typical AD
Hernandez et al. (2014)	4^b^	4/0	71.6	2.8	10.5	20.5	N.A.	F‐P or F‐T atrophy	N.A.	N.A.	AD pathology (+), Braak stage V‐VI
Ossenkoppele et al. (2015)	75^c^	51/24	65.8 ± 8.5	N.A.	15.5 ± 3.1	22.7	Executive > memory or visuospatial function	T‐P atrophy, similar to typical AD	mainly ^11^C‐PiB PET (+)	CSF total tau to Aβ1−42 ratio↑	Autopsy confirmed AD pathology (+) in 29 participants
Scialo et al. (2016)	1	0/1	68	4	16	27	Executive function	F atrophy	^18^F‐florbetapir PET (+)	CSF Aβ1−42↓, total tau↑	N.A.
Li et al., (2016)	1	0/1	71	4	12	12	Executive and memory	F‐T atrophy	^11^C‐PiB PET (+)	CSF Aβ1−42↓	N.A.
Kawakatsu, Kobayashi, and Hayashi (2017)	3	2/1	57.7	5	N.A.	N.A.	N.A.	Hippocampal and F atrophy	N.A.	N.A.	Amyloid (+), NFT (+)
Duclos et al. (2017)	1	0/1	61	4	16	N.A.	Executive, memory, social function	F‐T‐P atrophy	N.A.	CSF Aβ1−42↓, total tau↑, p‐tau↑	N.A.
Sawyer et al. (2017)	3	2/1	76.3	2.7	N.A.	N.A.	Executive function	F‐T or global atrophy	N.A.	N.A.	Amyloid (+), NFT (+)
Current study	1	1/0	66	7	13	10	Executive function > memory	Global atrophy with more severe on the right F‐T‐P area	^11^C‐PiB PET (+) ^18^F‐T807 PET (+)	Plasma Aβ1−42↑, Aβ1−40↑, total tau↑	N.A.

Abbreviations: ^11^CPiB: ^11^C‐labeled Pittsburgh compound B; AD: Alzheimer's disease; F: frontal; MMSE: Mini‐Mental State Examination; MRI: magnetic resonance imaging; N.A: not available; NFT: neurofibrillary tangles; NPT: neuropsychological test; P: parietal; PET: positron emission tomography; T: temporal.

a Selected based on poor executive function by neuropsychological tests.

b Brain AD pathology found in clinically diagnosed frontotemporal patients.

c Classified as behavioral variant (*n* = 46), dysexecutive variant (*n* = 20), or both (*n* = 9).

Clinical, neuroimaging, biomarker, and neuropathological characteristics of previously reported patients with fvAD are listed in Tables 3 and 4. A total of 188 patients from 18 reports were summarized in Tables 3 and 4. The mean age of onset was 61.8 ± 6.8 years, and 67% were men. All of them present with symptoms of frontal executive dysfunction and brain MRI showed global atrophy with emphasis on the frontoparietal or frontal–temporal atrophy without obvious asymmetry. Our index patient had a relatively early onset of executive symptoms (onset age less than 65 years old), which was compatible with those reported in the literature. Furthermore, although there was a trend showing a lower PiB ligand retention in the right hemisphere, which may be partly affected by the asymmetric right brain atrophy, the difference between hemispheres did not reach the statistical significance. Reviewing previously reported cases with fvAD (Tables 3 and 4), only three studies had the results of PiB‐PET scan. Among these three studies, two studies showed no gross differences between hemispheres from the provided images (Li, Zhou, Lu, Wang, & Zhang, 2016; Scialo et al., 2016) and one study did not measure the differences between hemispheres (Ossenkoppele et al., 2015). Notably, the brain MRI and FDG‐PET revealed asymmetrical atrophy and hypometabolism over right frontotemporal and parietal areas, which was corresponding to the increased retention of tau deposition and diffusely increased amyloid retention on the molecular imaging.

## 3 DISCUSSION

We describe here the clinical, neuropsychological, neuroimaging, and biofluid biomarker features of a patient with fvAD. The patient presented with progressive cognitive impairment associated with behavioral symptoms, mainly apathy, irritability, and agitation, followed by memory decline. Neuropsychological examination showed impairment in several cognitive domains, with prominent features of dysexecutive syndrome. Neuroimaging studies (Brain MRI and FDG‐PET) showed focal atrophy and hypometabolism in the corresponding areas, mimicking bvFTD. However, the molecular imaging study showed diffusely increased amyloid depositions by ^11^C‐PiB PET and retention of paired helical tau by ^18^F‐T807 PET (Chien et al., 2013), suggesting the evidence of brain amyloid and tau deposition observed in neuropathology findings of patients with AD. (Dubois et al., 2014). The plasma biomarker study, which showed elevated plasma Aβ1‐42, Aβ1‐42/Aβ1‐40 ratio, and total tau levels, was consistent with our previous findings in patients with AD (Chiu et al., 2012, 2013). Our patient with a behavioral‐predominant presentation fit the diagnosis of fvAD with both molecular imaging and biomarker evidence of AD pathology.

Despite the high prevalence of AD in the current aging society (Nichols et al., 2019), the exact proportion of frontal variants of AD is largely unknown. The description of this rare AD phenotype has been limited to case reports and small series (Alladi et al., 2007; Johnson, Head, Kim, Starr, & Cotman, 1999; Ossenkoppele et al., 2015; Scialo et al., 2016; de Souza et al., 2013). Recent series have suggested that fvAD might be misdiagnosed as FTD, accounting for 10%–30% of clinically diagnosed FTD patients by clinicopathological correlation, which may lead to underestimations of the true prevalence of fvAD (Forman et al., 2006; Mendez, Joshi, Tassniyom, Teng, & Shapira, 2013; Perry et al., 2017; Tan et al., 2017). Atypical variants of AD are reported to be associated with early‐onset age of presentation (<65 years old) but less so in the frontal than the posterior variant (Koedam et al., 2010; Mendez et al., 2012). One retrospective study has shown that among 125 patients with early‐onset AD, none had executive dysfunction as the main initial presentation, and the most common nonamnestic phenotype was associated with the language (26.4%) or visuospatial (28%) domain (Mendez et al., 2012). As described in the IWG‐2 criteria (Dubois et al., 2014), fvAD is defined as early, predominant, and progressive behavioral changes including association of primary apathy or behavioral disinhibition, or predominant executive dysfunction on cognitive testing. Memory decline tends to develop earlier and be more severe in fvAD than in FTD (Alladi et al., 2007; Mendez et al., 2013; Ossenkoppele et al., 2015; de Souza et al., 2013). Regarding behavioral symptoms, one retrospective study showed that patients with fvAD tend to show more frequent apathy, disinhibition, and loss of empathy and less perseverative/compulsive behavior or hyperorality compared to patients with bvFTD (Ossenkoppele et al., 2015). The same study also used “behavioral/dysexecutive” variant instead of “frontal” variant to address the relatively symmetric and insignificant atrophy in frontal lobes and some distinct clinical patterns between behavioral and dysexecutive forms. Motor presentations may also differ between fvAD and FTD because myoclonus tends to favor fvAD and early parkinsonism suggests FTD (Sawyer, Rodriguez‐Porcel, Hagen, Shatz, & Espay, 2017), consistent with our patient. Our patient fulfilled the presentation of fvAD in clinical aspects, although the asymmetric symptoms and neuroimaging findings mimicked FTD rather than AD.

The first pathological evidence of fvAD was reported in 1999 (Johnson et al., 1999), when three patients exhibited early and disproportionate impairments on frontal lobe function tests. Compared to a typical AD group, the “frontal AD” patients showed significantly higher paired helical tau containing NFTs but less amyloid plaque in the frontal cortex. This pattern was in concordance with molecular neuroimaging topography findings for our patient, for whom ^18^F‐T807 tau PET showed increased retention of tau in the right frontal region rather than ^11^C‐PiB amyloid PET, and was more correlated with clinical symptoms. In the past decade, amyloid PET imaging has been applied for clinical use and research measurement of Aβ burden, and the Aβ PET tracer retention is highly correlated with regional Aβ plaque density. Thus, amyloid imaging is useful in differentiating FTD from AD (Engler et al., 2008; Rabinovici et al., 2007, 2011), especially atypical variants of AD, although concerns persist that patients with FTD pathology may have coexisting false positivity for amyloid PET (Tan et al., 2017).

More recently, merging tau PET has shown value in diagnosis of AD and other tauopathy disorders (Chien et al., 2013; Shoup et al., 2013). In AD, tau PET imaging displays correlations with clinical symptoms and pathology, even in atypical variants (Ossenkoppele et al., 2016; Xia et al., 2017), which is consistent with findings for our index patient. Regarding structural images, it is suggested that fvAD still is associated with posterior (temporoparietal) atrophy similar to typical AD, but distinct from anterior (frontal) atrophy in bvFTD (Ossenkoppele et al., 2015). In our case, brain atrophy presents in both frontal and temporoparietal regions with an emphasis on the anterior part, again with intriguingly marked asymmetrical atrophy in the right hemisphere. This pattern could explain our patient's prominent apathy and behavior changes and relatively spared language functions with a right‐sided lesion.

More biofluid markers are becoming available for detecting underlying pathology in vivo and facilitating early diagnosis for proper treatment strategies. The CSF Aβ1‐42, Aβ1‐42/Aβ1‐40 ratio, and tau (especially phosphorylated tau) serve as biomarkers for AD (Blennow, Mattsson, Scholl, Hansson, & Zetterberg, 2015), whereas in FTD, only a nonspecific increase in tau is involved because of neurodegeneration. One study applied CSF biomarkers to support the diagnosis of fvAD in those previously clinically diagnosed as bvFTD (de Souza et al., 2013), but validated consensus blood‐based biomarkers for AD are still lacking. Our previous study using the highly sensitive IMR immunoassay to measure several AD‐related biomarkers from plasma showed that combined elevated plasma Aβ1‐42 and tau protein levels could differentiate mild cognitive impairment from AD with a sensitivity and specificity of 0.80 and 0.82, respectively (Chiu et al., 2013). Therefore, plasma biomarker profiles of our current patient also showed a consistent pattern, further supporting the diagnosis of AD. However, we did not have the conventional CSF biofluid markers for AD in our index patient, which is a limitation of the study.

In summary, we present a patient with the rare fvAD with initial presentation of asymmetrical frontal dysexecutive and behavioral problems, followed by memory decline and progression to moderate dementia. Characteristic asymmetric right‐sided predominant atrophy with brain hypometabolism and clinical symmetry broaden the phenotypes of fvAD. The application of integrated molecular imaging and biofluid markers is needed for proper diagnosis of this rare variant of AD.

## Data Availability

The data that support the findings of this study are available on request from the corresponding author. The data are not publicly available due to privacy or ethical restrictions.
